# Supplementary material for: mRNA processing in mutant zebrafish lines generated by chemical and CRISPR-mediated mutagenesis produces unexpected transcripts that escape nonsense-mediated decay
Source: PLoS Genet. 2017 Nov 21;13(11):e1007105. doi: 10.1371/journal.pgen.1007105 (PMC5716581; doi:10.1371/journal.pgen.1007105)
Supplement: S2 Fig — abca1asa9624 (ATP-binding cassette, sub-family A, member 1A), cd36sa30701 (cluster of differentiation cd), and pla2g12bsa659 (phospholipase A2 Group XIIB) yield PCR products (using pooled larvae from heterozygous incrosses) that match the predicted length of amplicons of the wildtype cDNAs and do not reveal a shorter amplicon expected with the omission of the affected exon (A). Modifying PCR conditions to look for evidence of a retained intron (i3–4) in pla2g12bsa659 did not yield a product in homozygous mutants. Band sizes of the ladders are shown in bases. B. Primers used and sizes anticipated are listed. C. Primer locations are indicated by arrows. The exons that are expected to be skipped as a result of ESS mutations are shown as dotted lines. For pla2g12bsa659, the splice acceptor sequence preceding exon 4 is mutated (and there is no downstream natural splice acceptor sequence). (PDF) [file pgen.1007105.s002.pdf]

A

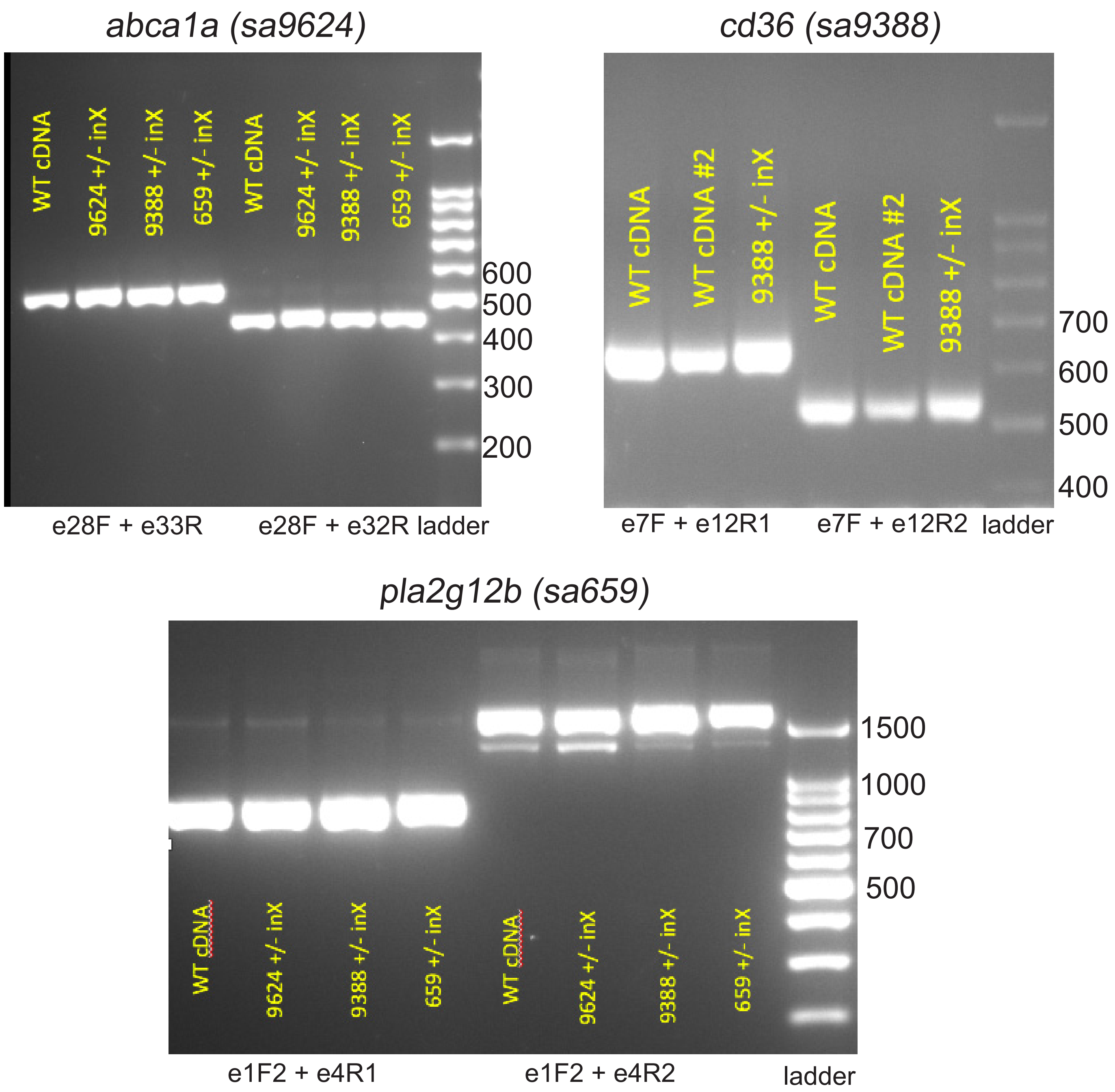

B

gene	primers used	expected size (bp)	expected altered size (bp)
abca1a	e28F+e33R	528	338
	e28F+e32R	437	247
cd36	e7F+e12R1	621	547
	e7F+e12R2	523	449
pla2g12b	e1F2+e4R1	763	597
	e1F2+e4R2	1517	1351

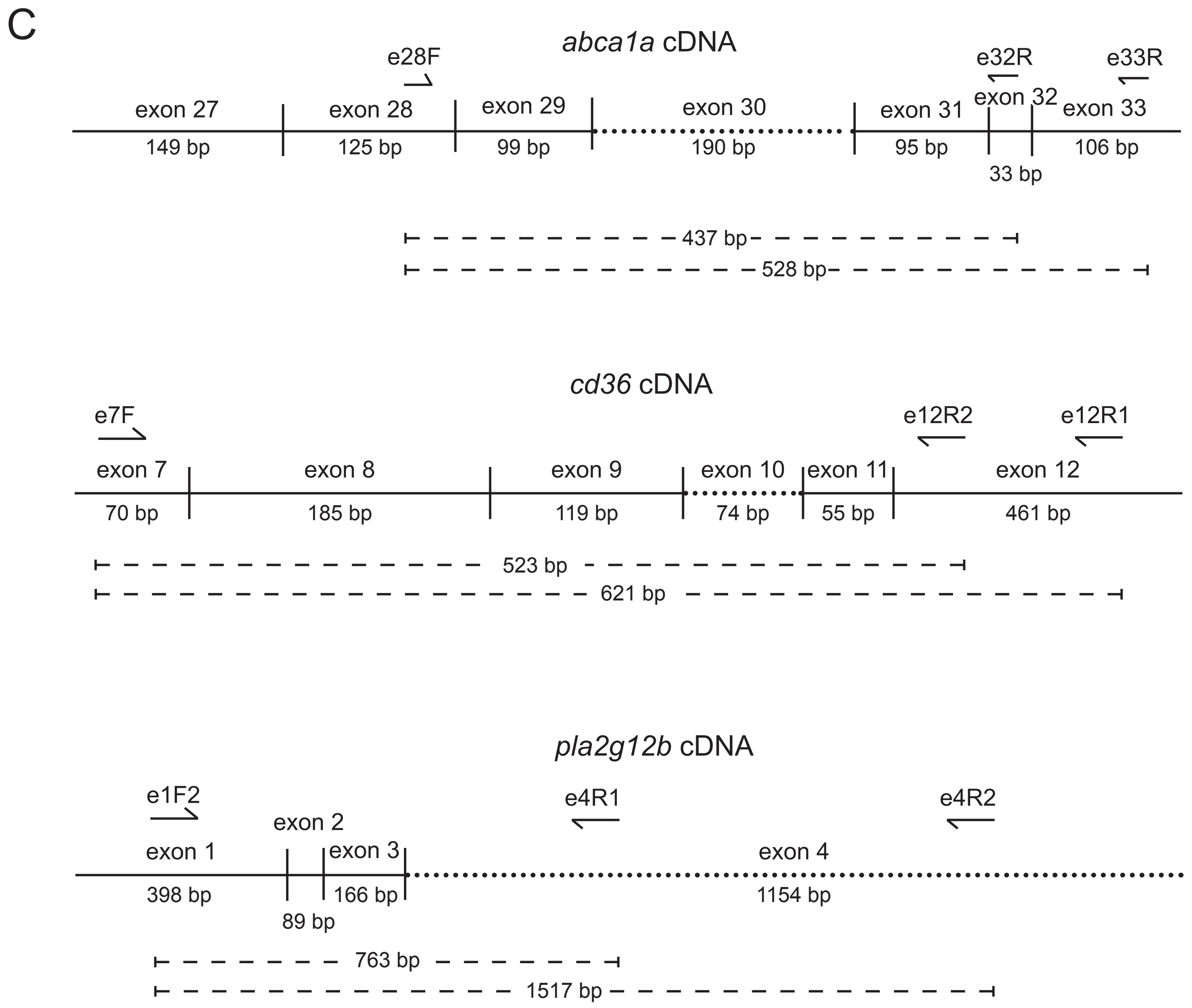
C
abca1a cDNA
e28F
e32R
e33R
exon 27
exon 28
exon 29
exon 30
exon 31
exon 32
exon 33
149 bp
125 bp
99 bp
190 bp
95 bp
33 bp
106 bp
437 bp
528 bp
cd36 cDNA
e7F
e12R2
e12R1
exon 7
exon 8
exon 9
exon 10
exon 11
exon 12
70 bp
185 bp
119 bp
74 bp
55 bp
461 bp
523 bp
621 bp
pla2g12b cDNA
e1F2
e4R1
e4R2
exon 1
exon 2
exon 3
exon 4
398 bp
89 bp
166 bp
1154 bp
763 bp
1517 bp

S2 Fig.
