# Supplementary material for: mRNA processing in mutant zebrafish lines generated by chemical and CRISPR-mediated mutagenesis produces unexpected transcripts that escape nonsense-mediated decay
Source: PLoS Genet. 2017 Nov 21;13(11):e1007105. doi: 10.1371/journal.pgen.1007105 (PMC5716581; doi:10.1371/journal.pgen.1007105)
Supplement: S5 Fig — The shaded yellow region indicates the skipped exon (2). For slc27a2, the shaded green region indicates the ATP/AMP motif and the shaded blue region indicates the FATP/VLACS motif [47]. For creb3l3a, the shaded green region indicates the basic region and the shaded blue region indicates the leucine zipper domain (identified in [48]). (PDF) [file pgen.1007105.s005.pdf]

```
slc27a2a_Dr    1     MLCPLLLGLLLSALLFLYIRFPFLPQDCAFALRTLNLGRLLARFGSR   47
slc27a2_Hs     1  MLSAIYTVLAGLLFLPLLVN-LCCPYFFQDIGYFLKVAAVGRRVRSYGKR   49
slc27a2_Mm     1  MLPVLYTGLAGLLLLPLLLT-CCCPYLLQDVRYFLRLANMARRVRSYRQR   49
slc27a2_Rv     1  MLPVLYTGLAGLLLLPLLLT-CCCPYLLQDVRFFLQLANMARQVRSYRQR   49
                  MLP LYT LAGLLLLPLL  Y CCPYL QD   FL  AN  R VRSY  R

slc27a2a_Dr   48  SPCFSTLERFAEVARKHPDKLFIVFGDERYTYRDADRISNRLANALRD--   95
slc27a2_Hs    50  RPARTILRAFLEKARQTPHKPFLLFRDETLTYAQVDRRSNQVARALHDHL   99
slc27a2_Mm    50  RPVRTILRAFLEQARKTPHKPFLLFRDETLTYAQVDRRSNQVARALHDQL   99
slc27a2_Rv    50  RPVRTILHVFLEQARKTPHKPFLLFRDETLTYAQVDRRSNQVARALHDHL   99
                  RP RTIL  FLE ARKTPHKPFLLFRDETLTYAQVDRRSNQVARALHDHL

slc27a2a_Dr   96  --RSGQIVALFHGNAPMYVFTWLALAKLGCTVALLNTNIRSRSLVHCCEC  143
slc27a2_Hs   100  GLRQGDCVALLMGNEPAYVWLWLGLVKLGCAMACLNYNIRAKSLLHCFQC  149
slc27a2_Mm   100  GLRQGDCVALFMGNEPAYVWIWLGLLKLGCPMACLNYNIRAKSLLHCFQC  149
slc27a2_Rv   100  GLRQGDCVALFMGNEPAYVWLWLGLLKLGCPMACLNYNIRAKSLLHCFQC  149
                  GLRQGDCVALFMGNEPAYVW WLGL KLGC MACLNYNIRAKSLLHCFQC

slc27a2a_Dr  144  SGAKTLITAAELVPAVLEVLQSLRQQQVSVLMLSGEAETQDIINLTNQVS  193
slc27a2_Hs   150  CGAKVLLVSPELQAAVEEILPSLKKDDVSIYYVSRTSNTDGIDSFLDKVD  199
slc27a2_Mm   150  CGAKVLLASPDLQEAVEEVLPTLKKDAVSVFYVSRTSNTNGVDTILDKVD  199
slc27a2_Rv   150  CGAKVLLASPELHEAVEEVLPTLKKEGVSVFYVSRTSNTNGVDTVLDKVD  199
                  CGAKVLL SPEL  AVEEVLP LKK  VSV YVSRTSNT G D  LDKVD

slc27a2a_Dr  194  RASEEAPPISLRQHITMKSPALYIYTSGTTGLPKAAVVTHEKVWMMSFLQ  243
slc27a2_Hs   200  EVSTEPIPESWRSEVTFSTPALYIYTSGTTGLPKAAMITHQRIWYGTGLT  249
slc27a2_Mm   200  GVSAEPTPESWRSEVTFTTPAVYIYTSGTTGLPKAATINHHRLWYGTGLA  249
slc27a2_Rv   200  GVSADPIPESWRSEVTFTTPAVYIYTSGTTGLPKAATINHHRLWYGTSLA  249
                   VS EP PESWRSEVTF TPA YIYTSGTTGLPKAA I H R WYGT L

slc27a2a_Dr  244  RLSGVCSSDIIYICLPLYHSAGFLAGLSGAIERGITVVLKSKFSASRFWD  293
slc27a2_Hs   250  FVSGLKADDVIYITLPFYHSAALLIGIHGCIVAGATLALRTKFSASQFWD  299
slc27a2_Mm   250  MSSGITAQDVIYTTMPLYHSAALMIGLHGCIVVGATLALRSKFSASQFWD  299
slc27a2_Rv   250  LRSGIKAHDVIYTTMPLYHSAALMIGLHGCIVVGATFALRSKFSASQFWD  299
                    SG  A DVIY T PLYHSAAL IGLHGCIV GAT ALRSKFSASQFWD

slc27a2a_Dr  294  DCREHNVTVIQYIGEVMRYLCNTPERENDRQHSVRLALGNGIRAETWREF  343
slc27a2_Hs   300  DCRKYNVTVIQYIGELLRYLCNSPQKPNDRDHKVRLALGNGLRGDVWRQF  349
slc27a2_Mm   300  DCRKYNVTVIQYIGELLRYLCNTPQKPNDRDHKVKKALGNGLRGDVWREF  349
slc27a2_Rv   300  DCRKYNATVIQYIGELLRYLCNTPQKPNDRDHKVKIALGNGLRGDVWREF  349
                  DCRKYNVTVIQYIGELLRYLCNTPQKPNDRDHKV  ALGNGLRGDVWREF

slc27a2a_Dr  344  LRRFGDVRVCECYGATEGNIGFFNYTGKIGSIGRVSAIHKMLFPYAFLKF  393
slc27a2_Hs   350  VKRFGDICIYEFYAATEGNIGFMNYARKVGAVGRVNYLQKKIITYDLIKY  399
slc27a2_Mm   350  IKRFGDIHVYEFYASTEGNIGFVNYPRKIGAVGRANYLQRKVARYELIKY  399
slc27a2_Rv   350  IKRFGDIHIYEFYASTEGNIGFMNYPRKIGAVGRENYLQKKVVRHELIKY  399
                   KRFGDI  YEFYA TEGNIGF NY RKIGAVGR NYLQKK   Y LIKY

slc27a2a_Dr  394  DPEKEEPVRGSDGLCVEAAPGETGLLVAKIHKLAPFEGYAKNSTQTEKKR  443
slc27a2_Hs   400  DVEKDEPVRDENGYCVRVPKGEVGLLVCKITQLTPFNGYAGAKAQTEKKK  449
slc27a2_Mm   400  DVEKDEPVRDANGYCIKVPKGEVGLLVCKITQLTPFIGYAGGKTQTEKKK  449
slc27a2_Rv   400  DVEKDEPVRDANGYCIKVPKGEVGLLICKITELTPFFGYAGGKTQTEKKK  449
                  DVEKDEPVRD NGYC  VPKGEVGLLVCKIT LTPF GYAG KTQTEKKK

slc27a2a_Dr  444  LRDVFQRGDMYFNTGDLILADRQGFLYFQDRIGDTFRWKGENVATTEVSE  493
slc27a2_Hs   450  LRDVFKKGDLYFNSGDLLMVDHENFIYFHDRVGDTFRWKGENVATTEVAD  499
slc27a2_Mm   450  LRDVFKKGDIYFNSGDLLMIDRENFVYFHDRVGDTFRWKGENVATTEVAD  499
slc27a2_Rv   450  LRDVFKKGDVYFNSGDLLMIDRENFIYFHDRVGDTFRWKGENVATTEVAD  499
                  LRDVFKKGD YFNSGDLLM DRENF YFHDRVGDTFRWKGENVATTEVAD
```

```
slc27a2a_Dr  494  ILLMLDCIEAANVYGVTVPGHEGRVGMAALQLTDGMEFDGSAAYEHMKNL  543
slc27a2_Hs   500  TVGLVDFVQEVNVYGVHVPDHEGRIGMASIKMKENHEFDGKKLFQHIADY  549
slc27a2_Mm   500  IVGLVDFVEEVNVYGVPVPGHEGRIGMASLKIKENYEFNGKKLFQHIAEY  549
slc27a2_Rv   500  IVGLVDFVEEVNVYGVPVPGHEGRIGMASIKMKENYEFNGKKLFQHISEY  549
                  IVGLVDFVEEVNVYGV VPGHEGRIGMAS K KEN EF GKKLFQHI  Y

slc27a2a_Dr  544  LPAYARPRFIRIQEELRLTGTFKQVKVQLVQEGFDPNSTRDRLFIMEENQ  593
slc27a2_Hs   550  LPSYARPRFLRIQDTIEITGTFKHRKMTLVEEGFNPAVIKDALYFLDDTA  599
slc27a2_Mm   550  LPSYARPRFLRIQDTIEITGTFKHRKVTLMEEGFNPTVIKDTLYFMDDAE  599
slc27a2_Rv   550  LPSYSRPRFLRIQDTIEITGTFKHRKVTLMEEGFNPSVIKDTLYFMDDTE  599
                  LPSYARPRFLRIQDTIEITGTFKHRKVTL EEGFNP VIKD LYFMDD

slc27a2a_Dr  594  QTFVPLTEEIFSAITAGRTRL  614
slc27a2_Hs   600  KMYVPMTEDIYNAISAKTLKL  620
slc27a2_Mm   600  KTFVPMTENIYNAIIDKTLKL  620
slc27a2_Rv   600  KTYVPMTEDIYNAIIDKTLKL  620
                  KT VPMTE IYNAI  KTLKL
```

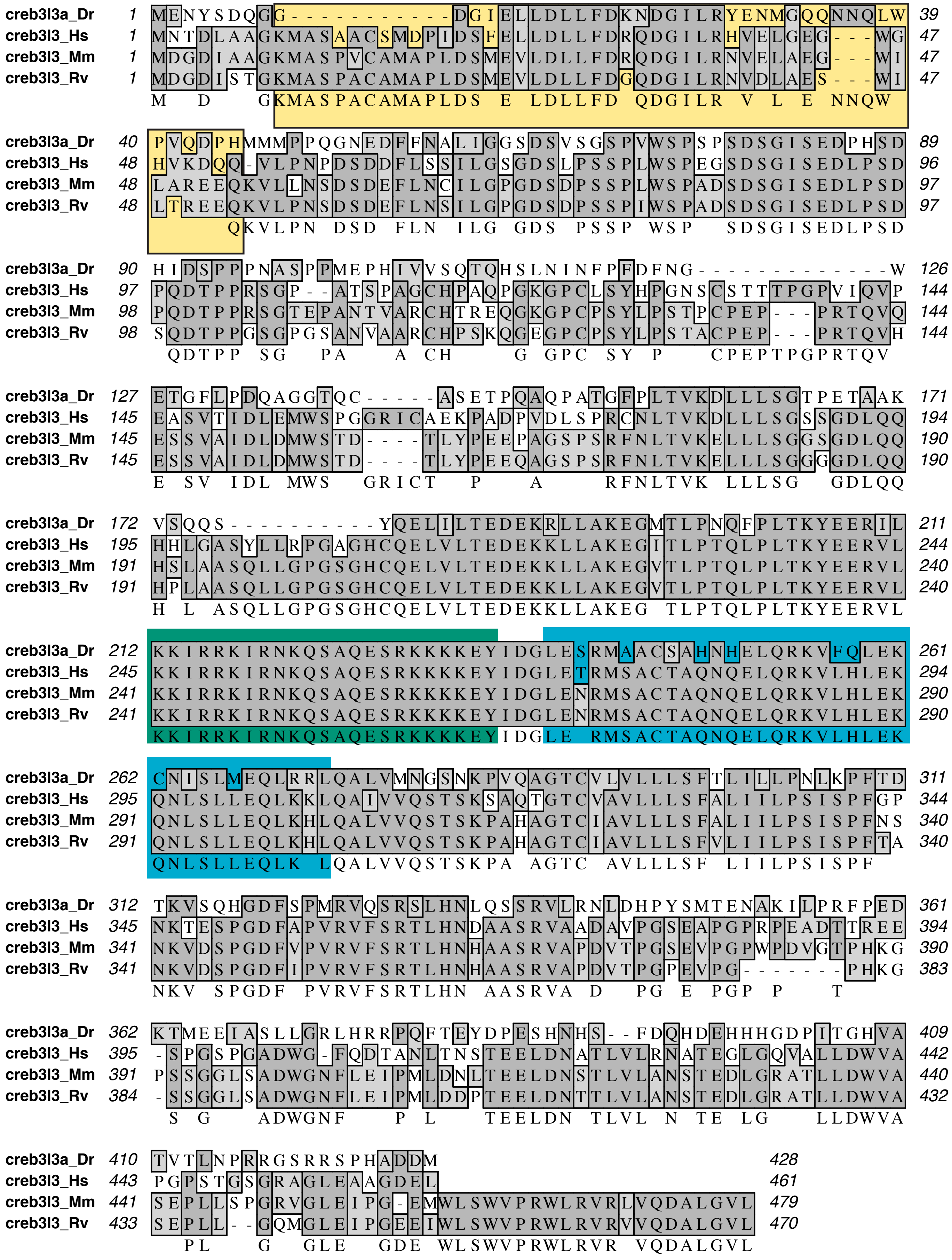
creb3l3a_Dr 1 MENYSDQGG-----------DGIELLDLLFDKNDGILRYENMGQQNNQLW 39
creb3l3_Hs 1 MNTDLAAGKMASAACSMDPIDSFELLDLLFDRQDGILRHVELGEG---WG 47
creb3l3_Mm 1 MDGDIAAGKMASPVCAMAPLDSMEVLDLLFDRQDGILRNVELAEG---WI 47
creb3l3_Rv 1 MDGDISTGKMASPACAMAPLDSMEVLDLLFDGQDGILRNVDLAES---WI 47
M D GKMASPACAMAPLDS E LDLLFD QDGILR V L E NNQW
creb3l3a_Dr 40 PVQDPHMMMPPQGNEDFFNALIGGSDSVSGSPVWSPSPSDSGISEDPHSD 89
creb3l3_Hs 48 HVKDQQ-VLPNPDSDDFLSSILGSGDSLPSSPLWSPEGSDSGISEDLPSD 96
creb3l3_Mm 48 LAREEQKVLLNSDSDEFLNCILGPGDSDPSSPLWSPADSDSGISEDLPSD 97
creb3l3_Rv 48 LTREEQKVLPNSDSDEFLNSILGPGDSDPSSPIWSPADSDSGISEDLPSD 97
QKVLPN DSD FLN ILG GDS PSSP WSP SDSGISEDLPSD
creb3l3a_Dr 90 HIDSPPPNASPPMEPHIVVSQTQHSLNINFPFDFNG-------------W 126
creb3l3_Hs 97 PQDTPPRSGP--ATSPAGCHPAQPGKGPCLSYHPGNSCSTTTPGPVIQVP 144
creb3l3_Mm 98 PQDTPPRSGTEPANTVARCHTREQGKGPCPSYLPSTPCPEP---PRTQVQ 144
creb3l3_Rv 98 SQDTPPGSGPGSANVAARCHPSKQGEGPCPSYLPSTACPEP---PRTQVH 144
QDTPP SG PA A CH G GPC SY P CPEPTPGPRTQV
creb3l3a_Dr 127 ETGFLPDQAGGTQC-----ASETPQAQPATGFPLTVKDLLLSGTPETAAK 171
creb3l3_Hs 145 EASVTIDLEMWSPGGRICAEKPADPVDLSPRCNLTVKDLLLSGSSGDLQQ 194
creb3l3_Mm 145 ESSVAIDLDMWSTD----TLYPEEPAGSPSRFNLTVKELLLSGGSGDLQQ 190
creb3l3_Rv 145 ESSVAIDLDMWSTD----TLYPEEQAGSPSRFNLTVKELLLSGGGGDLQQ 190
E SV IDL MWS GRICT P A RFNLTVK LLLSG GDLQQ
creb3l3a_Dr 172 VSQQS----------YQELILTEDEKRLLAKEGMTLPNQFPLTKYEERIL 211
creb3l3_Hs 195 HHLGASYLLRPGAGHCQELVLTEDEKKLLAKEGITLPTQLPLTKYEERVL 244
creb3l3_Mm 191 HSLAASQLLGPGSGHCQELVLTEDEKKLLAKEGVTLPTQLPLTKYEERVL 240
creb3l3_Rv 191 HPLAASQLLGPGSGHCQELVLTEDEKKLLAKEGVTLPTQLPLTKYEERVL 240
H L ASQLLGPGSGHCQELVLTEDEKKLLAKEG TLPTQLPLTKYEERVL
creb3l3a_Dr 212 KKIRRKIRNKQSAQESRKKKKEYIDGLESRMAACSAHNHELQRKVFQLEK 261
creb3l3_Hs 245 KKIRRKIRNKQSAQESRKKKKEYIDGLETRMSACTAQNQELQRKVLHLEK 294
creb3l3_Mm 241 KKIRRKIRNKQSAQESRKKKKEYIDGLENRMSACTAQNQELQRKVLHLEK 290
creb3l3_Rv 241 KKIRRKIRNKQSAQESRKKKKEYIDGLENRMSACTAQNQELQRKVLHLEK 290
KKIRRKIRNKQSAQESRKKKKEYIDGLE RMSACTAQNQELQRKVLHLEK
creb3l3a_Dr 262 CNISLMEQLRRLQALVMNGSNKPVQAGTCVLVLLLSFTLILLPNLKPFTD 311
creb3l3_Hs 295 QNLSLLEQLKKLQAIVVQSTSKSAQTGTCVAVLLLSFALIILPSISPFGP 344
creb3l3_Mm 291 QNLSLLEQLKHLQALVVQSTSKPAHAGTCIAVLLLSFALIILPSISPFNS 340
creb3l3_Rv 291 QNLSLLEQLKHLQALVVQSTSKPAHAGTCIAVLLLSFVLIILPSISPFTA 340
QNLSLLEQLK LQALVVQSTSKPA AGTC AVLLLSF LIILPSISPF
creb3l3a_Dr 312 TKVSQHGDFSPMRVQSRSLHNLQSSRVLRNLDHPYSMTENAKILPRFPED 361
creb3l3_Hs 345 NKTESPGDFAPVRVFSRTLHNDAASRVAADAVPGSEAPGPRPEADTTREE 394
creb3l3_Mm 341 NKVDSPGDFVPVRVFSRTLHNHAASRVAPDVTPGSEVPGPWPDVGTPHKG 390
creb3l3_Rv 341 NKVDSPGDFIPVRVFSRTLHNHAASRVAPDVTPGPEVPG-------PHKG 383
NKV SPGDF PVRVFSRTLHN AASRVA D PG E PGP P T
creb3l3a_Dr 362 KTMEEIASLLGRLHRRPQFTEYDPESHNHS--FDQHDEHHHGDPITGHVA 409
creb3l3_Hs 395 -SPGSPGADWG-FQDTANLTNSTEELDNATLVLRNATEGLGQVALLDWVA 442
creb3l3_Mm 391 PSSGGLSADWGNFLEIPMLDNLTEELDNSTLVLANSTEDLGRATLLDWVA 440
creb3l3_Rv 384 -SSGGLSADWGNFLEIPMLDDPTEELDNTTLVLANSTEDLGRATLLDWVA 432
S G ADWGNF P L TEELDN TLVL N TE LG LLDWVA
creb3l3a_Dr 410 TVTLNPRRGSRRSPHADDM 428
creb3l3_Hs 443 PGPSTGSGRAGLEAAGDEL 461
creb3l3_Mm 441 SEPLLSPGRVGLEIPG-EMWLSWVPRWLRVRLVQDALGVL 479
creb3l3_Rv 433 SEPLL--GQMGLEIPGEEIWLSWVPRWLRVRVVQDALGVL 470
PL G GLE GDE WLSWVPRWLRVR VQDALGVL

S5 Fig.
