# Supplementary figures and images for: mRNA processing in mutant zebrafish lines generated by chemical and CRISPR-mediated mutagenesis produces unexpected transcripts that escape nonsense-mediated decay
Source: PLoS Genet. 2017 Nov 21;13(11):e1007105. doi: 10.1371/journal.pgen.1007105 (PMC5716581; doi:10.1371/journal.pgen.1007105)

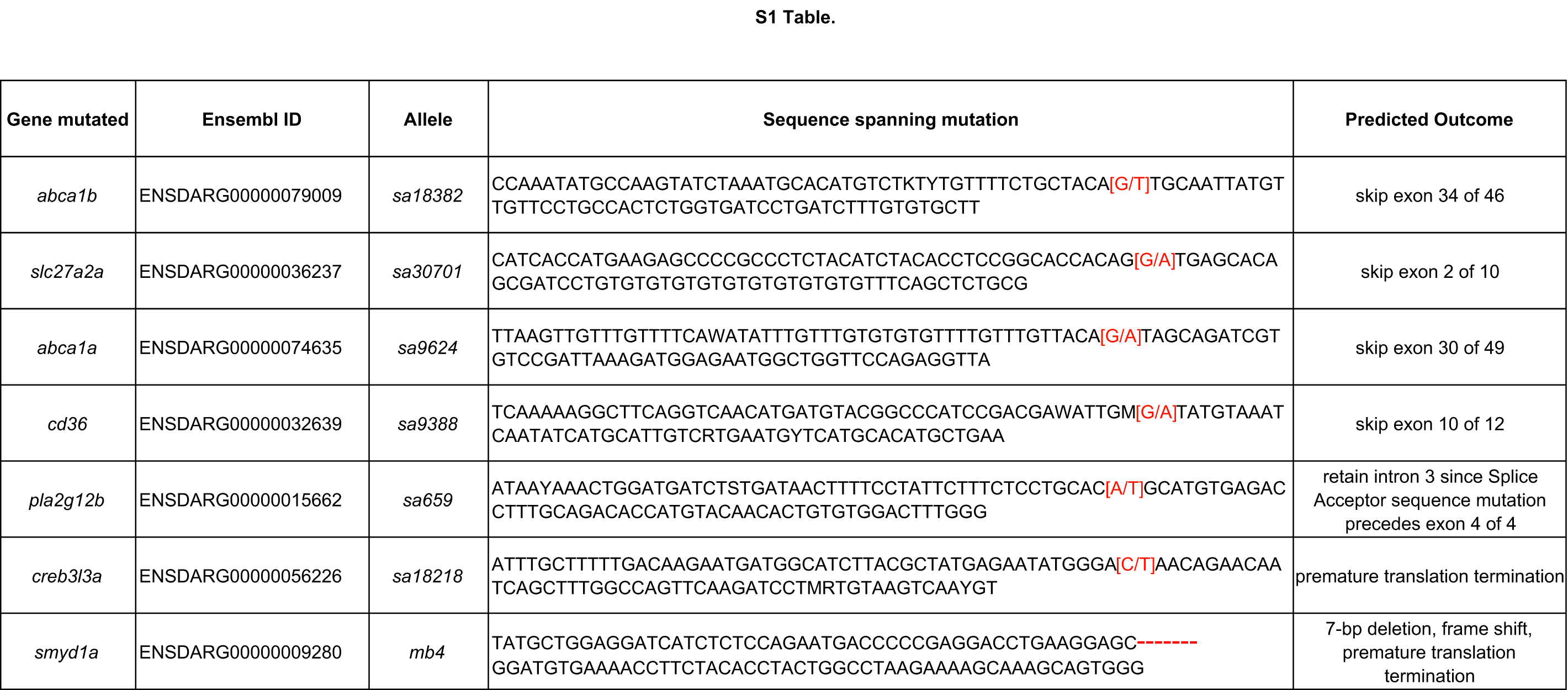

Supplement: S1 Table — (TIF) [file pgen.1007105.s006.tif]

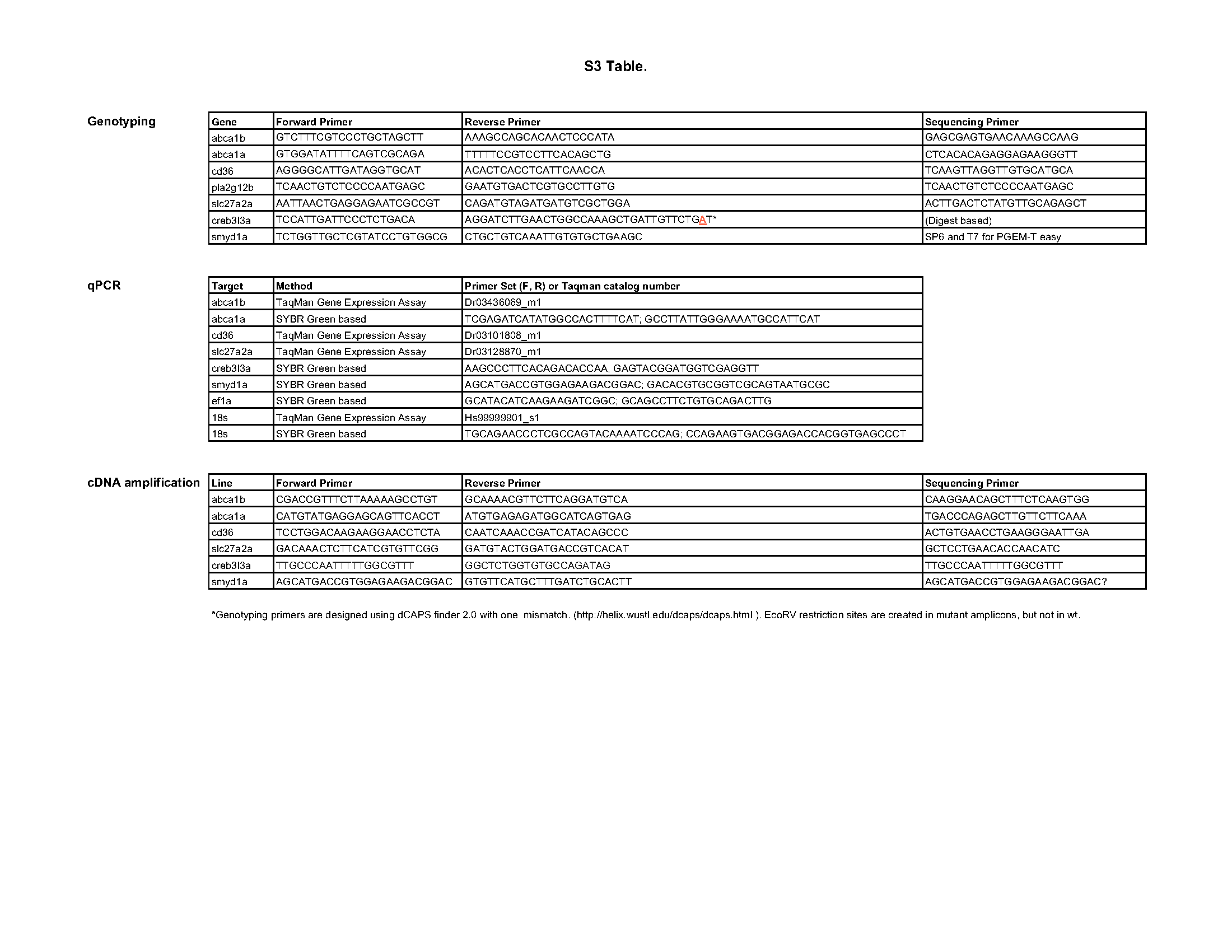

Supplement: S3 Table — (TIFF) [file pgen.1007105.s008.tiff]
